# Sweet sorghum as biofuel feedstock: recent advances and available resources

**DOI:** 10.1186/s13068-017-0834-9

**Published:** 2017-06-08

**Authors:** Supriya Mathur, A. V. Umakanth, V. A. Tonapi, Rita Sharma, Manoj K. Sharma

**Affiliations:** 1 Crop Genetics & Informatics Group, School of Biotechnology, Jawaharlal Nehru University, New Delhi, India; 2 Indian Council of Agricultural Research-Indian Institute of Millets Research, Hyderabad, India; 3 Crop Genetics & Informatics Group, School of Computational and Integrative Sciences, Jawaharlal Nehru University, New Delhi, India

**Keywords:** Sorghum, Biofuel, Sorghum breeding, Genomics, Feedstock, Grass genetics

## Abstract

Sweet sorghum is a promising target for biofuel production. It is a C4 crop with low input requirements and accumulates high levels of sugars in its stalks. However, large-scale planting on marginal lands would require improved varieties with optimized biofuel-related traits and tolerance to biotic and abiotic stresses. Considering this, many studies have been carried out to generate genetic and genomic resources for sweet sorghum. In this review, we discuss various attributes of sweet sorghum that make it an ideal candidate for biofuel feedstock, and provide an overview of genetic diversity, tools, and resources available for engineering and/or marker-assisting breeding of sweet sorghum. Finally, the progress made so far, in identification of genes/quantitative trait loci (QTLs) important for agronomic traits and ongoing molecular breeding efforts to generate improved varieties, has been discussed.

## Background

Burning of fossil fuels has led to significant increase in the global carbon dioxide (CO_2_) concentrations, which in turn is contributing to the global warming with extreme changes in climate and weather, worldwide [1]. Besides, fossil fuels are not going to last forever [2–4]. Progress in the technology for fuel recovery and discovery of new fossil fuel reserves may extend the depletion timeline of fossil fuels but the capacity of our planet to combat the catastrophic effects of their combustion is exhausting fast. Apparently, there is an urgent need to explore the sustainable energy sources, which can not only fulfill our energy needs but more importantly mitigate the adverse impact on the environment.

Biofuels are sustainable and renewable source of energy derived from organic matter in the form of biomass. Biofuels can be derived from plant as well as animal biomass. Studies showed that plants grown for biofuel purposes have potential to reduce the net greenhouse gas emissions. Schmer and colleagues [5] reported that usage of corn and switchgrass as source of biofuels reduced the greenhouse gas emissions by −29 to −396 g of CO_2_ equivalent per mega joule of ethanol per year. Currently, about 2.5% of the world’s transportation fuels are produced from the crop plants including maize, sugarcane, and vegetable oils [6]. However, both maize and sugarcane are input-intensive food crops. Extensive usage of these crops as biofuel feedstock will not only threaten food security but would also compete with other food crops for irrigation and arable land resources. Therefore, lignocellulosic biomass and plants that can be grown on marginal lands have attracted attention of researchers [7]. In addition to the agricultural waste, several grasses like switchgrass, *Miscanthus,* and foxtail millet have been identified as candidate bioenergy feedstock. However, since domestication of these grasses is relatively recent, targeted efforts to develop genetic and genomic resources for them that can eventually be used for their improvement are underway [8–15]. Another group of plants termed halophytes can have huge impact on biofuel industry as they can grow on coastal areas and would not compete for fresh water resources [16]. However, efficient conversion of lignocellulosic biomass to fermentable sugars largely depends on the pretreatment of cell walls which, due to their complex structural organization, are naturally recalcitrant to efficient deconstruction. Therefore, several parallel studies have been carried out to understand the mechanism of cell wall biosynthesis and degradation and identify candidates for reducing cell wall recalcitrance using model systems, rice and *Arabidopsis* [17–22]. An alternate solution to overcome these challenges is to utilize the grasses (for example, sugarcane and sweet sorghum) where sugars, accumulated in the form of juice, can be easily extracted and directly fermented to produce ethanol.

*Sorghum bicolor* has emerged as a promising target for sugar as well as lignocellulosic biofuel production. It has relatively low input requirements with ability to grow on marginal lands. Cultivated varieties of sorghum exhibit diverse phenotypic and morphological traits. Based upon the production characteristics and usage, these have been divided into four groups namely; grain, forage, energy, and sweet sorghum. Grain sorghum varieties are three to six feet tall with large ear heads and primarily serve as food for humans or livestock feed. The coarse fast-growing forage sorghum varieties are utilized for feed, silage, and grazing [23]. Energy sorghum is specifically bred for high lignocellulosic biomass that can be converted to biofuels, whereas sweet sorghum, also known as sweet stalk sorghum, refers specifically to genotypes that accumulate soluble sugars in the stalk [24]. Sweet sorghum may grow up to twenty feet tall and produce significantly higher biomass yields compared to grain sorghum. Stems of sweet sorghum are thicker and fleshier than the grain varieties, though the seed yield is relatively low [25].

Due to high sugar content and ease of extractability, sweet sorghum is one of the leading feedstock crops for new-age biofuels and focus of this review. The sugar concentration in sweet sorghum stalks is measured in Brix units, which represents the percent soluble sugars. One-degree Brix is equal to 1 g of sugar per 100 g of juice. The Brix content varies in different varieties and also depends on the environmental conditions, internode position, time of the year, and stage of harvesting [26]. Sweet sorghum can accumulate juice up to 78% of the total biomass, whereas the Brix content of sweet sorghum has been estimated to range from 14 to 23% [27, 28]. The sugars in sweet sorghum stalks mainly comprise sucrose (~75%) with some amount (~2.6%) of fructose and glucose [29]. In comparison to lignocellulosic biomass crops like switchgrass and *Miscanthus*, soluble sugars in the form of glucose, fructose, and sucrose in sweet sorghum are readily fermentable [30]. Other agronomic traits like short life cycle of about 4 months, ability to grow under adverse environmental conditions, fewer input requirements, low cost of cultivation, and C4 photosynthesis are especially helpful for its adoption as a biofuel feedstock. Different sweet sorghum cultivars exhibit differential effect of salinity on seed germination and seedling growth [31]. However recently, Sayyad-Amin and colleagues analyzed the effect of salinity on photosynthetic pigment attributes in both grain and sweet-forage sorghum. Their results, at both, vegetative and reproductive stages, suggested that sorghum can possibly be irrigated using saline water up to 150 mM NaCl [32]. Furthermore, C4 photosynthesis is particularly important as it contributes to higher nitrogen and water use efficiency as well as overall robustness of sweet sorghum making it better equipped to survive in the dry regions with higher light intensity/temperatures [33, 34]. Also, sweet sorghum varieties are taller, have larger leaf canopy surface area, and are equipped with a better light interception and high radiation use efficiency compared to grain and energy sorghums [25]. According to U.S. Department of Agriculture, the ratio of energy invested to energy obtained during biofuel extraction from sweet sorghum is estimated as 1:8 [35], which may further be improved using engineering and molecular breeding technologies. Ethanol produced from sweet sorghum is safer for environment due to low sulfur content, low biological, and chemical oxygen demand and high octane rating [36]. Although, annual ethanol output from sweet sorghum depends on several factors including genetic background, time of the year, soil quality, and other environmental factors, sweet sorghum crop is estimated to produce up to 8000 l/ha/year of ethanol [37].

**Fig. 1:**
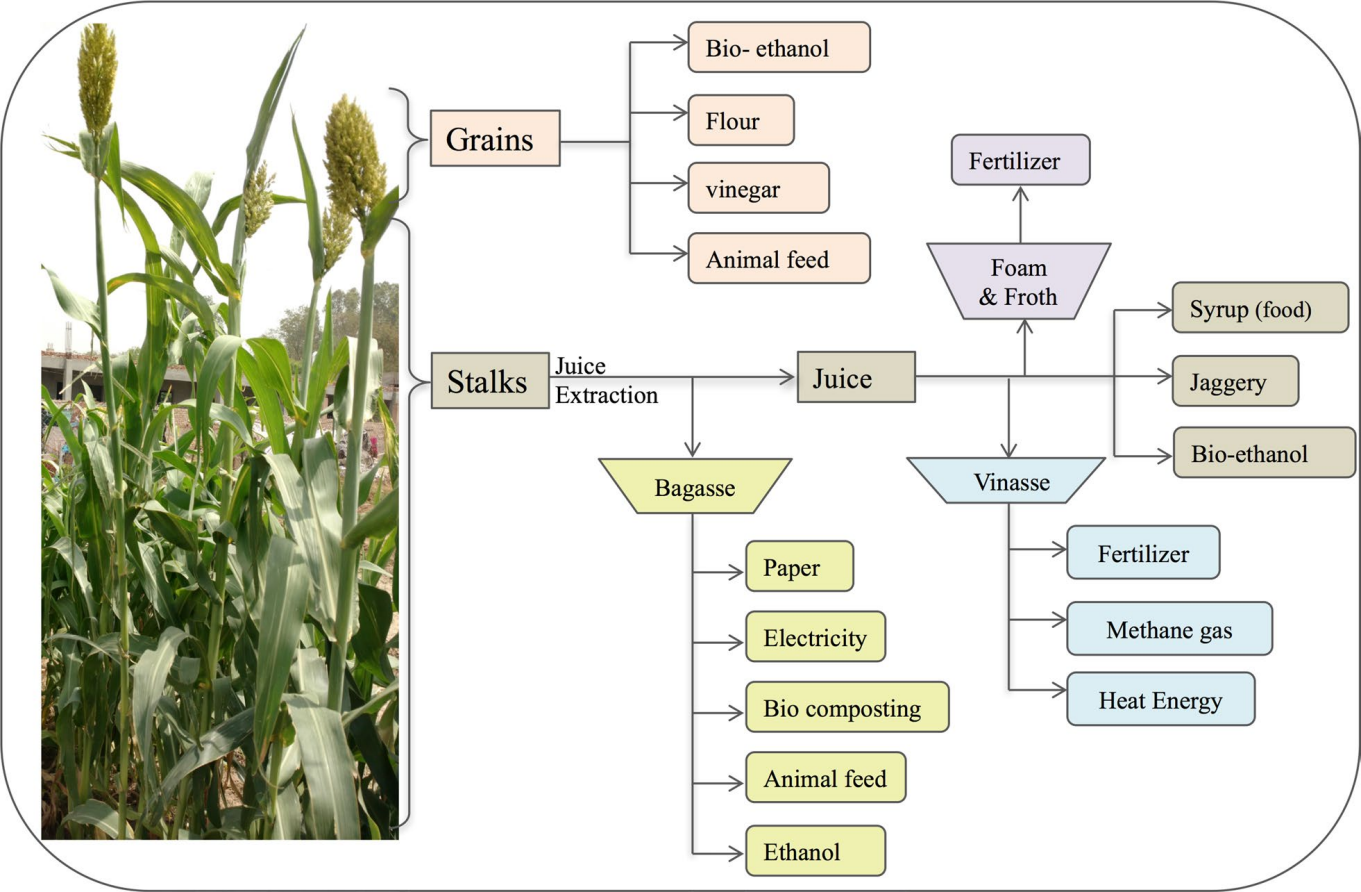
Sweet sorghum as a multipurpose crop. The various uses of sweet sorghum juice, grains, and other byproducts have been illustrated

In addition to the stem sugars that are major commodity for sweet sorghum cultivation, co-products in the form of grains, bagasse, vinasse, steam, foam, and froth are also utilized as raw material for range of purposes (Fig. 1). Syrup obtained from the juice extracted from the stalk of the plant has been used as a sweetener in America since 1890s [35]. In India, the juice is mainly used to make syrup and jaggery [38], though its usage for cooking and lighting fuel has also been explored. Nimbkar Agricultural Research Institute (NARI) in rural Maharashtra, India, developed a lantern-cum-stove that uses low-grade ethanol developed from sweet sorghum and provides energy for lightening as well as clean fuel for cooking [39]. For biofuel purposes, juice is fermented to ethanol that can be used as a replacement for conventional fuels. During concentration of juice to syrup, the foam and froth produced can be processed and used to feed livestock or as an organic fertilizer [40]. After juice extraction, the fibrous leftover material, known as bagasse, serves as a raw material for handmade paper, electricity generation, and bio-composting [40, 41]. The lignocellulosic biomass in the form of bagasse can also be used for ethanol production and biodegradable plastics. The silage, derived from bagasse, is rich in micronutrients and minerals and hence, is a nutritious source of animal feed especially for the dairy cattle. Even, the liquid distillate left after extraction of ethanol from sweet sorghum juice, called vinasse or stillage, is used as a fertilizer in agricultural fields that abates the problem of waste disposal [40]. Other uses of vinasse are anaerobic digestion to produce methane gas for combustion to produce heat energy. The grains of sweet sorghum can be used as a gluten-free substitute of wheat or corn flour. Although starch reserves in grains can also be used for ethanol and vinegar production; poor quality grain is mostly used for the animal feed [40].

Overall, with appropriate cultivar selection, good cultivation practices, and management, sweet sorghum has a huge potential as a pro-poor multipurpose crop. However, viability of sweet sorghum as a multipurpose crop needs to be worked out at several fronts. The sweet sorghum germplasm exhibits tradeoffs between sugar content and biomass yields with some genotypes containing high sugar content with lower biomass, while others usually with lower sugar yields have high stalk biomass [42]. The ideal genotypes would have these two traits combined, i.e., higher biomass with high sugar yields. Overall, sweet sorghum improvement programs are motivated by three major goals including (1) improving the quantity and quality of the stalk juice, (2) identification of multipurpose varieties that can accumulate sugars in the stalk as well as produce good quality grains and high biomass, and (3) engineering resistance to combat potential biotic and abiotic stresses. Meeting these goals not only requires extensive germplasm screening but also informed breeding efforts, genetic and genomic resources, optimization of plant transformation and engineering strategies, cross utilization of information from other closely related species, and a well-defined strategy. Here, in this review, we provide an overview of existing resources available for sweet sorghum research and highlight the recent advances made to initiate crop improvement efforts.

## Life cycle and growth conditions

Sweet sorghum is an annual plant with a short life cycle of about 4 months. It allows two crops per year though optimal planting date varies with the place of cultivation and the variety [43]. It is a warm-season crop with the highest productivity in rainy and summer seasons. Sweet sorghum is mainly adapted to arid and semi-arid regions, with temperature range of 12–37°C, optimum range being 32–34°C [44]. Yield of sweet sorghum is directly affected by the planting time. In the semi-arid tropical climate, ideal time for planting sweet sorghum is early June to early July [45]. Loam and sandy loam soils with soil temperature above 18°C and pH around 5.8 are considered best for the optimum growth and maximum stem juice yield [46]. Although increased seeding rate compromises the size of individual plants and total yields, it has positive impact on the total biomass and sugar yields [47, 48]. Tillage and use of fertilizers can also significantly affect the total yields. Pittelkow and colleagues evaluated several environmental and agronomic factors on no-till yields [49]. Their results showed that under water limiting conditions, no-till system increases overall yield as compared to conventional tillage systems in arid regions. It has also been reported that sweet sorghum requires ~36% of nitrogen fertilizer that is needed for similar ethanol yields from corn [50, 51]. However, the use of moderate amount of nitrogen fertilizers enhances sweet sorghum growth rate and ethanol yields [47, 52].

Although moisture availability is critical for the plant growth [53], sweet sorghum is relatively drought-tolerant and can be adapted to grow on marginal lands with low water availability [54, 55]. The well-developed root structure that can extend up to 2 m below ground aids to obtain moisture from the soil. Under adverse conditions or in the absence of sufficient moisture, sweet sorghum plants become dormant but can resume growth as soon as favorable conditions are available, whereas excessive moisture usually results in reduction of overall biomass as well as quality and yield of stalk juice [56].

**Fig. 2:**
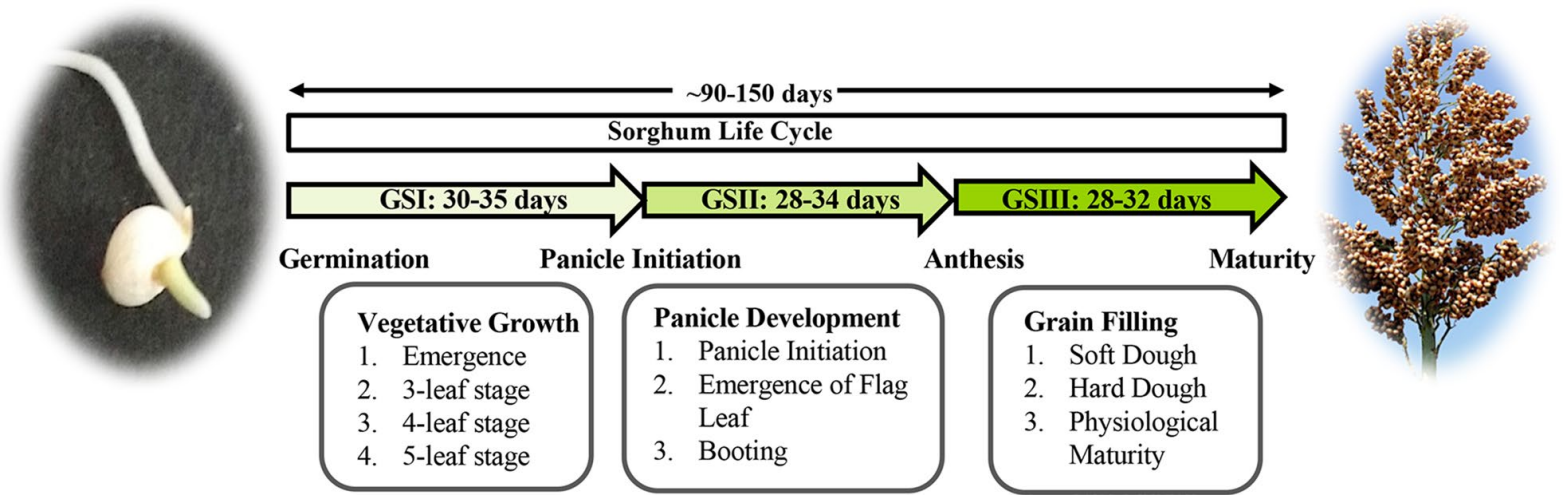
Growth phases and stages during sorghum life cycle. The key developmental stages and growth phases during sorghum life cycle have been illustrated

The life cycle of sorghum has been divided into three distinct growth phases with ten morphologically distinguishable growth stages [57]. The first phase involves germination to panicle initiation (GS1); second phase starts with panicle initiation and ends with the anthesis (GS2); and the third phase starts from anthesis until maturity (GS3). Morphologically distinguishable growth stages include emergence, 3-leaf stage, 5-leaf stage, panicle initiation, flag leaf stage, booting, half bloom, soft dough, hard dough, and physiological maturity. Duration from emergence to flowering in tropical sweet sorghum varieties usually ranges from about 55 to 70 days; however, this phase is quite variable in different varieties. Especially, in the varieties adapted to temperate climate zones, this phase can be further extended by 20–30 days beyond what is reported for tropical varieties [44, 58]. Flowering is directly influenced by photoperiod though sensitivity to photoperiod varies among different varieties of sweet sorghum [40]. Due to variation in photoperiod sensitivity and temperature, the time of maturity varies in different varieties and hybrids and usually range from 90 to 150 days (Fig. 2).

Accumulation of soluble sugars in sweet sorghum stems is reported to surge after the internode elongation stops at the time of anthesis. Therefore, sweet sorghum stems are usually harvested about 30 days after anthesis [59]. However, stage of maximum sugar accumulation varies in different varieties with some genotypes mainly accumulating sugars between dough stage and physiological maturity, whereas others accumulate sugars up to 15 days post-physiological maturity [60]. Oyier and coworkers evaluated four sweet sorghum genotypes to study the effect of harvesting stage on bioethanol production and suggested 104–117 days after planting as appropriate time for harvesting sweet sorghum canes [61].

## Origin, genetic diversity, and breeding

*Sorghum bicolor* (L.) Moench is a member of Andropogoneae tribe of subgroup panicoideae of the grass family, poaceae [40, 62]. The genus *Sorghum* is divided into five subgenera including *Sorghum*, *Stiposorghum*, *Chaetosorghum*, *Heterosorghum*, and *Parasorghum*. The subgenus *Sorghum* contains three species including *S. bicolor*, *S. propinquum*, and *S. halepense*. Further, *S. bicolor* has three subspecies including *S. bicolor*, *S. bicolor drummondii*, and *S. bicolor verticilliflorum* (formerly referred as *arundinaceum*) [40, 63, 64].

Further, based on the grain shape, glume, and panicle, cultivated varieties of *Sorghum bicolor* have been classified into five basic races including bicolor, guinea, caudatum, kafir, and durra [63]. Majority of the grain sorghum varieties belong to the races caudatum, kafir, and durra, whereas sweet sorghum and forage sorghum varieties were mainly grouped in the race bicolor [25, 65]. However, later studies showed clustering of sweet sorghum lines with other *S. bicolor* genotypes suggesting that sweet sorghum has a polyphyletic origin and therefore, apart from race bicolor, may have parentage from other previously mentioned races as well [66]. In Africa, where most of the wild germplasm has originated, intermediate varieties are also common. For instance, there are many durra-bicolor intermediates in Ethiopian highlands [67]. Race kafir has contributed to many intermediate varieties in Tanzania and regions of South Africa.

Sweet Sorghum is widely cultivated in USA, Brazil, India, China, Mexico, Sudan, Argentina, and many other countries in Asia and Europe. Like grain sorghum, it has its origin in Africa [40] but migration routes from Africa to other parts of the world and its emergence as a specific variety of *S. bicolor* are not clear. The highest genetic and phenotypic diversity in both wild and cultivated accessions of sorghum are found in the central Africa [68]. Many natural variants and hybrid cultivars suited to diverse agro-climatic conditions worldwide have been developed using conventional breeding technologies. According to an estimate, more than 4000 cultivars of sweet sorghum are cultivated all over the world [37]. The breeding methods used for sweet sorghum improvement include introduction, pedigree selection, and backcrossing as short-term improvement programs, whereas population improvement has been used as a long-term strategy for simultaneous improvement of economic traits [44]. Recently, a comprehensive survey of all the resources encompassing mutant populations, QTL dissection, identification, and isolation of genes controlling important agronomic traits, that are necessary for advancing molecular breeding and deeper understanding of the system, has been reported [69, 70].

In United States, sweet sorghum was introduced in the form of Chinese Amber (from china), Orange, Sumac/Redtop, Gooseneck/Texas Seeded Ribbon Cane, Honey and White African (from China and Africa via France) [71]. United States Department of Agriculture (USDA) uses National Plant Germplasm System and the database Germplasm Resources Information Network (GRIN)-Global to manage national resource of plant germplasm. It hosts the botanical and agronomic information of 52,575 accessions of *Sorghum bicolor* (L.) Moench subsp. *bicolor* [72]. A collection of 2180 accessions of sweet sorghum in the US National Plant Germplasm System has served as a source of germplasm for developing varieties in the Mediterranean region and Latin America [73]. Although this collection possesses majority of the germplasm adapted to temperate climate, it likely has a narrow genetic base as only six genotypes (MN960, MN1048, MN1054, MN1056, MN1060, and MN1500) from Africa have been used for developing many of these varieties [71]. Early breeding efforts in USA were concentrated on using sweet sorghum as a sugar crop. Although several sweet sorghum breeding programs have been initiated in United States, most of the varieties in cultivation were developed at the U.S. Sugar Crops Field Station at Meridian, Mississippi. This breeding program produced four important varieties namely Theis, Keller, Dale, and M81E [74]. All the four varieties give high yield of syrup per ton of the stalk. Recently, Leite and colleagues (2017) evaluated 45 genotypes for association among agro-industrial traits for ethanol yield and prioritized several lines including BR500R, BR505R, CMSXS633R, and CMSXS634R that showed positive association with ethanol yield and are therefore, promising candidates for breeding purposes [75].

Inbred lines are important to ensure availability of genetically uniform individuals with heritable desired traits (like sugar content), which can be further used for the development of elite lines or hybrids. In hybrid development program, two types of inbred lines are required namely female inbred lines (A/B lines) and male inbred lines (R lines) [76]. Female inbred lines with high sugar content were released by Texas A&M University [74]. The combining ability of the parental lines and hybrids has recently been used to select parental lines for future crossing strategies and screen the hybrids for commercial cultivation [77].

Some parts of the central and southern region, subtropical regions of Uttar Pradesh, and Uttaranchal are most suitable for commercial cultivation of sweet sorghum in India [78]. Most of the sweet sorghum cultivars available in India have been developed by Indian Council of Agricultural Research (ICAR)–Indian Institute of Millets Research (IIMR; formerly known as Directorate of Sorghum Research) and All India Coordinated Research Project (AICRP) centers for Sorghum. International Crops Research Institute for Semi-Arid Tropics (ICRISAT) has a large repository for *S. bicolor* (L.) Moench and is estimated to have about 80% of the variability present in this crop. It has a total of 39,234 accessions from 93 countries [79]. The various genotypes of sorghum at ICRISAT gene bank have been divided into seven collections namely, accession collection, conversion collection, cultivar collection, genetic stock collection, basic collection, wild and weedy sorghums, and core collection [80]. In addition to safeguarding the genetic diversity, ICRISAT makes these accessions freely available to researchers at other institutions.

Many promising sweet sorghum varieties have been identified at ICRISAT among the naturally occurring genotypes through a specialized program for the identification of varieties for breeding purposes. Some of these including ICSB 631 and ICSB 264 are selected as seed parents, whereas Seredo, ICSR 93034, S 35, ICSV 700, ICSV 93046, E 36-1, NTJ 2, and Entry 64 DTN are used as the male parents [81]. Under the All India Research Improvement Project (AICRP) on Sorghum, several improved varieties have been released by IIMR and other AICRP centers using pedigree method. These include SSV 74, SSV 84, CSV 19SS, and CSV 24SS [44]. Several cultivars and hybrid varieties, that were developed at IIMR and ICRISAT, are being evaluated at national level, while many are ready for commercial cultivation [78]. CSH 22SS is the most popular hybrid of sweet sorghum that was developed at IIMR and produce high sugar yields. It is used as a bench mark for evaluating the performance of new test cultivars [78]. Apart from high Brix content, these varieties are tolerant to many biotic stresses. Such tolerance abrogates losses due to pest injury and microbial infections. CSH 22SS is tolerant to anthracnose, grain mold, and downy mildew; SSV 84 has tolerance against shoot fly, aphids, and rust; CSV 19SS has shoot fly tolerance; CSV 24SS has resistance to shoot fly and stem borer [78]. Many lines resistant to stem borer infection have also been identified. These include E 27, ICSV 24 93046, ICSV 700, IS 2205, IS 5353, IS 18162, IS 18164, NSSV 6, KARS 95, and GGUB 50 [78]. Most of these varieties are mainly adapted for rainy season and can be grown during summer, provided lifesaving irrigations are available. Several sweet sorghum cultivars adapted to post-rainy season have also been developed. These include SPSSV 30, SPSSV 11, SPSSV 20, SPSSV 40, and SSV 74. Further, varieties that perform consistently across rainy and post-rainy seasons have been identified in these trials. According to a recent AICRP annual report, 16 hybrids and 23 varieties are being evaluated at various locations [82]. Apart from breeding for important traits like juice content, biomass yield, and stress tolerance, trials for environment-specific varieties are in progress through multi-location and on-farm testing [78]. AICRP centers are located at several locations in India. These locations are used to evaluate sorghum varieties and hybrids for several agronomic traits under different environmental conditions. Several hybrids including SPH 1713, DMS 8A × RSSV76, DMS 26A × SSV 74, DMS 30A × SSV 74, and varieties like SPV 2074 have been developed that give superior ethanol yields as compared to CSH 22SS. These varieties have been reported to have higher Brix content, juice content, and grain yields. Other hybrids that are being evaluated and are reported to outperform CSH 22SS include ICSA 560 × IS 17814, ICSA 560 × IS 21991, and RS 1220A × SSV 74. Some environment/region-specific sweet sorghum cultivars have also been released for commercial cultivation that include RVICSH 28 (Madhya Pradesh) and Phule Vasundhara (Maharashtra).

At NARI, indigenous germplasm collections (forage and grain varieties) were crossed with exotic lines (American Germplasm) to identify superior germplasm with features like high cane yield and high Brix percentage [28]. Among 22 accessions, which were evaluated for juice quality, stalk and grain yields, and total energy production per unit land area, S 21-3-1 and S 23-1-1 were the best performers and are therefore, promising candidates. Hybrids including Madhura, NARI-SSH45, and NARI-SSH48 with good grain yield and high Brix content have also been developed at NARI [28].

China is another major center of diversity and producer of sorghum in Asia. Chinese sorghum is also called kaoliang. The major sorghum producing areas include northern and northeastern regions of the country. A well-characterized sorghum germplasm collection including sweet sorghum varieties has also been established [83]. The approaches used for breeding of sweet sorghum cultivars in china are introduction and breeding by selection, utilization of heterosis, cross breeding, induced mutation breeding, and transgenic breeding [84, 85]. Some of the sweet sorghum varieties/hybrids developed in China include Shennong No. 2 [85] and Liaotian 1 by Liaoning AAS in 1997 [84]. The sweet sorghum hybrid Shennong No. 2 was developed by sweet sorghum breeding group of Shenyang University by heterosis using ROMA and ATx623 as parent lines. Shennong No. 2 surpassed its both parents in dry matter production [86]. Other sweet sorghum varieties/hybrids that are grown in China on large scale include M81E, Lvneng No. 2, 3, Nengsi No. 1 and hybrids Chuntian No. 2, Liaotian No. 1, 2, and Nengsiza No. 1 [86]. Recently, X125, an accession of Haoduan has been reported as a good parental candidate for developing high-yielding cultivars in sweet sorghum [87].

France, Italy, and Germany are the main centers of sweet sorghum research in European Union. In 2009, European Union initiated an international project titled “SWEETFUEL” that was aimed to improve the sorghum cultivars for better yields. In addition to European countries, Brazil, India, Mexico, and South Africa were partners in this consortium. Major objectives were to understand the biology of sugar accumulation; effect of drought stress and plant phenology on sugar yields; investigate the technological, environmental, economic, and social aspects for long-term sustainability; optimization of cultural practices; and crop modeling. As an outcome of SWEETFUEL project, several recommendations have been made that include harvesting stems with leaves and grains, using grains of sweet sorghum for ethanol production, sweet sorghum cultivation on low carbon soils and designing ethanol plants for the full utilization of leaves as well as the surplus bagasse [88]. Overall, the results of SWEETFUEL project suggested sweet sorghum as a strategic complementary crop to sugarcane in tropical climates, whereas cold tolerance remains a major constraint in temperate areas. In a recent study, a preliminary field trial with two sweet sorghum hybrids (ICSSH31 and Bulldozer) revealed differential photochemical acclimation to cold in these hybrids opening new avenues for selecting traits to broaden growing season of sorghum ideotypes in temperate climates as well [89]. Mocoeur and colleagues used a recombinant inbred line, derived from a cross between a sweet and a grain sorghum, to test the stability and genetic controls of fifteen morphological, biomass, and biofuel traits under temperate maritime and continental conditions [58]. Their results suggest that the tall and fast maturing sorghum plants with high Brix content have high potential for breeding as biofuel crop in Northern Europe.

## Molecular markers, genome sequence, and DNA polymorphisms

Most of the genetic mapping studies in sorghum are based on grain sorghum varieties mainly BTx623. The marker systems developed for sorghum have been extensively reviewed elsewhere [90]. Briefly, these include RFLPs (restriction fragment length polymorphism), AFLPs (amplified fragment length polymorphism), STS (sequence-tagged sites), DArTs (Diversity Array Technology), SSRs (simple sequence repeats), and PAVs (presence absence variations) [91–96]. Mace and colleagues constructed a linkage map of sorghum where authors integrated six independent sorghum component maps to generate a consensus map [97]. The component maps were based on SSRs, AFLPs, and high-throughput DArT markers. The consensus map consisted of 1997 markers mapped to 2029 unique loci spanning 1603.5 cM. The average marker density in the map was 1 marker/0.79 cM. This map is currently serving as the genetic resource for mapping in sorghum research. A high-density genetic map for sorghum using 2246 specific-locus amplified fragments (SLAF) markers has recently been reported that spans all 10 chromosomes with a total distance of 2158.1 cM [98].

Elucidation of polymorphic genetic loci in sweet sorghum through various marker systems is also gaining momentum. Ritter and colleagues studied the genetic diversity between grain and sweet sorghum cultivars through AFLP markers [66]. Their results suggested the polyphyletic origin for sweet sorghum, i.e., sweet sorghum-specific traits have evolved several times independent of each other. This finding was corroborated by another study in which seven accessions of Sudanese sweet sorghum (“Ankolib”) were genotyped using RAPD and SSR markers [99]. The most important finding of the study was distant relationship of one accession named Bengaga to the other six accessions. Unlike the others accessions, Bengaga has juicy stems and good quality seeds that can be used to produce flour. Presence of this feature, independent of the other related sweet sorghum accessions, indicates polyphyletic origin. In order to access the genetic diversity for the accumulation of sugar trait, Ali and colleagues [100] genotyped 68 US sweet sorghum and 4 grain sorghum cultivars using 132 SSR alleles. Authors identified diverse sweet sorghum accessions, which were polymorphic at marker loci with significant difference in sugar content. These polymorphic marker loci can be used for mapping sugar content-related genes in sweet sorghum. Despite having diverse origin, sweet sorghum lines could be distinguished into separate groups based on usage (biofuel or syrup) through genetic markers. Using AFLP and SSR markers, Pecina-Quintero et al. [101] grouped six sweet sorghum lines into two distinct groups based upon their uses. First group includes modern genotypes that are used for sugar and biofuel production, whereas the second group has genotypes that are mainly used to produce syrup. In 2013, Wang and colleagues [102], reported genotyping of 142 parent lines of sweet sorghum using SSR markers. Although the study could not correlate marker-based analysis with agronomic traits, it provided information about selection criteria for parent lines for sweet sorghum hybrid breeding. In the same year, Billot and Colleagues [103] published a survey of 3367 sorghum accessions using SSR markers and generated a reference set, which is very helpful in identification, classification, setting up breeding programs, and investigations related to biological understanding of sorghum plant.

The genome of sorghum is estimated to be ~730 Mb, organized into ten chromosomes. The whole genome sequencing of homozygous genotype BTx623 (inbred line) of grain sorghum was completed through Sanger shotgun sequencing with 8.5-fold coverage [104]. Subsequently, new sequence data and assemblies were added and used to improve annotations. The current release (v3.1) of the sorghum genome is available at the Phytozome genome portal of Joint Genome Institute [105]. Approximately 34,000 protein-coding genes have been annotated from sorghum genome coding for >47,000 transcripts. Very recently, McCormick and colleagues have reported an improved assembly as well as annotation of sorghum genome, as preprint version on bioRxiv (http://biorxiv.org/content/early/2017/02/21/110593: Accessed on April 7, 2017).

The sorghum genome information is also hosted at Plant Genome and Systems Biology (PGSB) [106]. This database provides sequence information as well as comparative viewer to compare syntenic regions in sorghum with that of rice and *Brachypodium*. Ensembl Plants is another cyber infrastructure developed as a part of European Plant Genomics infrastructure and hosts genomic data for various plant species. It also hosts the sorghum genomic data, assembly, annotation, and comparative genomic information using sequence data produced by JGI [107]. Similarly, Sorghum Transcription Factor Database provides sequence information for about 1826 predicted transcription factors loci belonging to 56 families in *S. bicolor* [108]. Because of significant microcolinearity between sorghum, rice, and *Brachypodium* genomes [8], tools developed for rice/*Brachypodium* [19, 109] can serve as an important framework to strengthen the functional genomic studies in sorghum.

The whole genome sequencing of sweet sorghum is still awaited. However, large amount of data has been generated by differential hybridization using microarrays and resequencing to explore the genetic variation and sequence polymorphisms in grain and sweet sorghum cultivars [100, 110, 111]. Calvino and coworkers used Affymetrix sugarcane GeneChip^®^ arrays to identify DNA polymorphisms in grain and sweet sorghum varieties, BTx623 and Rio, respectively, by comparing the differences in the hybridization intensities [111]. Authors identified 30 candidate genes differentially expressed between sweet and grain sorghum with single-nucleotide polymorphisms. Zheng et al. [112] sequenced two sweet sorghum lines (Keller and E-Tian) and one grain sorghum inbred line (BTx623) to determine genetic variations in their genomes and identified >1 million SNPs, ~99,000 indels, and more than 17,000 copy number variations between sweet and grain sorghums. Authors shortlisted 1442 genes, mainly belonging to metabolism pathways of sugar/starch, nucleic acids, lignin, DNA damage, etc., that differentiate tested grain and sweet sorghum cultivars. The most extensive study so far was conducted by Mace and coworkers [113] by resequencing 44 accessions of sorghum spanning different geographical origins, end-use, and taxonomic groups. They identified more than 4.9 million SNPs and 1.9 million indels from the re-sequenced genomes. Leveraging such datasets, SorGSD (http://sorgsd.big.ac.cn/]) has been developed that provides a web-based query interface to search SNPs in sorghum accessions [114]. It contains about 62 million SNPs from 48 re-sequenced sorghum accessions that includes improved varieties, landraces, weedy accessions, and wild species collected from various parts of the world. These data can serve as a very useful resource for genotyping large populations, marker-assisted selection, and molecular mapping. The SorGSD also provides the links to other genome and transcriptome databases available for sorghum research.

## Transcriptomics and gene regulation

High-throughput transcriptomic technologies such as microarrays and RNAseq have revolutionized the scope and scale of gene expression analysis in plants, and sorghum is no exception. Affymetrix designed first commercially available sorghum GeneChip^®^, SorghWTa520972F (https://www.ncbi.nlm.nih.gov/geo/query/acc.cgi?acc=GPL17576) that carries 1,026,373 probes for 149,182 exons from 27,577 genes. In addition to annotated genes, it also carries probes representing putative non-coding RNAs, small RNAs, chloroplast, and mitochondrial genes. Shakoor and coworkers [115] used these arrays for expression analysis of four vegetative tissues including shoots, roots, leaves, and stems from six diverse genotypes of grain (R159), sweet (Fermont & Atlas), forage (PI152611), and bioenergy sorghum (PI455230 & AR2400). Tissue and genotype-specific expression of genes highlighted the significance of inter and intraspecific variation in sorghum. Conversely, Agilent Technologies Ltd. developed customized DNA arrays comprising 28 and 44K features for sorghum. Johnson and colleagues [116] used Agilent 28K arrays to analyze changes in gene expression in response to individual or combined heat and drought stresses in grain sorghum, whereas 44K arrays of sorghum have been used to investigate genetic variation and expression diversity between grain (BTx623) and sweet sorghum (Keller) lines [117].

RNA sequencing is now gaining popularity due to potential to reconstruct the whole genome from the transcriptomic data. Dugas et al. [118] used RNAseq to investigate the gene expression in response to osmotic stress and abscisic acid stress in sorghum. Chopra et al. [119] performed RNAseq with profile of contrasting cold responsive genotypes to identify differentially expressed genes in response to cold stress, whereas Sui and coworkers [120] compared transcript profiles of two sweet sorghum lines, M81E (salt tolerant) and ROMA (salt sensitive) to evaluate response to salt stress and corresponding increase in sugar content. Recently, Fracasso and colleagues [121] compared the transcriptomic profile of a drought-tolerant (IS22330) and sensitive sweet sorghum plant (IS20351) using RNAseq and reported constitutively high expression of drought response related genes in the tolerant cultivar.

Furthermore, an excellent resource of cDNA clones has been generated for sorghum by coupling RNA sequencing data from spikelet, stem, and seed tissues with functional annotations derived from a cDNA library [122]. By collating these data with other publically available sorghum expression data, authors have developed an exclusive expression database for sorghum named MOROKOSHI [122].

On similar lines, Nakamura and coworkers analyzed the publically available expression data and constructed a database (CATchUP; http://plantomics.mind.meiji.ac.jp/CATchUP) to provide information about the expressed genes [123]. In fact, expression data for sorghum have also been integrated on the phylogenomic database, Phytozome (https://phytozome.jgi.doe.gov/).

Further, with advent of small RNA sequencing, the differential accumulation and role of microRNAs in sugar accumulation in sweet sorghum is beginning to unfold. Several miRNA families have been identified that show varied expression in grain and sweet sorghums [124] and may be important for regulation of sugar accumulation. Characterization of stem-specific miRNA identified from sweet sorghum cultivars would shed more light on this unexplored territory [125].

Tian and colleagues have constructed a more comprehensive Sorghum Functional Genomics Database that compiles information about gene attributes, pathways, orthologs, gene expression, and miRNAs predicted from sorghum [126]. Authors have also provided tools to construct networks of genes based on the co-expression, predicted protein–protein interactions, miRNA-target pairs, and a GBrowse to visualize the SNPs. The classification and data for eight super families including transcription factors/regulators, protein kinases, monolignol biosynthesis-related enzymes, R-genes, cytochrome 450, ubiquitins, organelle-genes, and carbohydrate-active enzymes has also been integrated in the SorghumFDB. Comparative phylogenomic analysis of several gene families with important roles in regulating agronomic traits has led to identification of several important candidates for functional studies in sorghum [9, 127, 128]. Several small-scale studies have also been carried out to characterize the expression divergence mainly of sugar metabolizing, transport, and storage enzymes associated with sugar accumulation in sweet sorghum cultivars [26, 129, 130]. Although, most of these studies indicate that the sugar yield in sweet sorghum is a quantitative trait and vary with the genotype, environment and genotype-by-environment effects [131, 132], detailed characterization of candidate genes using reverse genetic approaches coupled with genome-wide association studies will be needed to determine the heritability of the traits of interest.

## In vitro regeneration and genetic transformation of sweet sorghum

Genetic transformation and engineering is a promising technology to investigate the gene functions and generate improved cultivars at a rapid rate. Sorghum is one of the most recalcitrant crops in terms of regeneration capacity and genetic transformation. However, significant progress has been made in optimizing the regeneration procedures and transformation systems for grain and sweet sorghum in the recent past [133–140].

For establishing a successful transformation pipeline, there are three essential prerequisites. These include (a) an optimized regeneration system, (b) an efficient genetic transformation method, and (c) a robust strategy for selection of putative transformants.

### Regeneration system

Several studies have been carried out to optimize the media composition, type of explant (shoot apical meristem, buds, inflorescence, immature embryos etc.), and supplements for regeneration media. The basal media that have been used in sweet sorghum callus induction and/or regeneration include Murashige and Skoog (MS), Linsmaier & Skoog (LS), and Gamborg. The most common formulation used for callus induction is MS basal medium with 2 mg/l 2,4-d, 0.5–1 mg/l kinetin, and 3% sucrose [135, 139, 141, 142]. Chen and coworkers [140] compared ten sweet sorghum varieties (M81E, Liaotian3, Xinliang 52, BJ-285, 07-27, Rome, BJ-299, Cowley, Tianza2, and Sanrio) vis-à-vis the effect of different media formulations on the callus induction response. The study compared three different formulations of MS, B_5_, and N_6_ basal medium salts for callus induction and regeneration. In addition, proline, sucrose, and 2,4-d were used in these media. Callus induction media were designated as MSI (MS), MBI (MS + B_5_), and NBI (N_6_ salts + B_5_) for induction. Similarly, regeneration media are designated as MSR, MBR, and NBR. For callus induction, all the three combination produced same efficiency. However, for regeneration, MBR and MSR produced almost similar regeneration frequencies but none of the tested genotypes regenerated on NBR medium indicating MS as the most appropriate basal medium. Out of ten varieties tested, Xinliang 52 had the highest callus induction, whereas 07–27 showed the highest regeneration frequency. Further, as suggested by Sharma and colleagues [143], removing leaf-like structures from calli on the regeneration medium can enhance regeneration efficiency and may be helpful to enhance shoot regeneration from proliferating calli. Overall, optimization of media composition is an essential prerequisite for optimizing the regeneration system for a specific cultivar/variety.

Genotype, source, and physiological state of the explants also play a major role in the regeneration and transformation efficiency [133–137, 144]. The explants tested for sweet sorghum regeneration include immature/mature embryos, immature inflorescence, shoot tips, segments of primordial leaves, and hypocotyl segments from in vitro seedlings [135, 144]. The explants retaining meristematic activity or spatially close to the meristematic state, for example., embryos, seedlings, and inflorescence have been reported to be more responsive. Immature embryos are most widely used for embryogenic callus formation and are shown to give highest transformation efficiency [137, 138]. The source of embryos also has a significant impact on transformation efficiency. Zhao and coworkers [145] reported that embryo explants harvested from field-grown sorghum plants resulted in better transformation frequency as compared to greenhouse produced embryo explants. However, harvesting immature embryos is very tedious, and their availability is also very limited. Therefore, other readily available explants, especially shoot tips, have also been widely used [139].

As reported for several other crops, genotype also directly affects the morphology and frequency of embryogenic calli [140]. Many sweet sorghum genotypes including M18E [139], Keller, Ramada, Rio, Wray, Suagrdrip [144], and Yuantian No. 1 [139] have been evaluated for their potential to regenerate through embryogenic callus. Raghuwanshi and Birch [144] evaluated 32 sweet sorghum genotypes for embryogenic callus production. Among these, Ramada was the most successful cultivar with 89% callus induction on M11 medium (modified MS + sucrose + B5).

Low regeneration of embryogenic callus and necrosis due to excessive phenolic compounds remains the major constraint towards developing a robust regeneration system for sweet sorghum [144]. The explants with genotypes that produce lower amount of phenolics during callus formation have better survival rate through regeneration phase. Further, addition of antioxidants like PVP (Polyvinylpyrrolidone) [139], coconut water [146], activated charcoal [147], l-proline, and l-asparagine [148] have been used to reduce the concentration of toxic phenolics. Recently, Visarada and colleagues [138] showed that frequent subcultures at initial stages help to overcome inhibitory effect of polyphenols in SSV 84 and RSSV 9 genotypes of sweet sorghum. However, the regeneration response towards different combinations of cytokines and auxins or other additives also varies with the genotype of the explant.

### Methods for genetic transformation

Particle bombardment as well as *Agrobacterium*-mediated transformation has been used to optimize the transformation of sorghum [137, 144, 149–151]. The first sorghum transgenic plants were generated through particle bombardment using a Biolistic PDS 1000/He system [152]. A resting period of 1 week after particle bombardment has been shown to improve the transformation efficiency in some of the sweet sorghum genotypes [138]. Recently, Raghuwanshi and colleagues [144] reported optimization of transformation procedure for sweet sorghum using particle bombardment and immature embryo as the explant. However, the transformation efficiency achieved was only ~0.01% per excised embryo.

Zhao and coworkers [145] optimized *Agrobacterium*-mediated transformation in sorghum with an average transformation efficiency of ~2%. Since then, several sorghum varieties have been transformed through *Agrobacterium*-mediated transformation procedures and transformation efficiency has also improved [145, 153]. Basu and coworkers [154] used shoot apical meristems for genetic transformation through *Agrobacterium*-mediated transformation. They altered the expression of genes encoding for caffeoyl-CoA-O-methyltransferase and Caffeic acid-O-methyltransferase through antisense gene cassette and generated the transgenic sweet sorghum plants with reduced lignin content. Li and colleagues [155] used this system to introduce a *Bt cry1Ah* gene in sweet sorghum varieties, BABUSH and MN-3025, and optimized an average transformation efficiency of 2.38%. Wu and colleagues [136] have reported optimization of highly efficient *Agrobacterium*-mediated transformation procedure where transformation efficiency up to 33% has been achieved. However, the transformation efficiency is largely determined by the genotype of the source plant. Several *Agrobacterium* strains like LAB4404, AGL1 have been used for sorghum transformation, but LBA4404 is the most successful and frequently used strain for sorghum transformation [136, 145].

Prolonged co-culturing with the bacteria or high inoculum of bacterial cells during *Agrobacterium*-mediated transformation of callus compromises the health of explants/calli as well as regeneration efficiency, whereas the addition of various concentrations of cefotaxime, antibiotic cephalosporin, and the amino acids asparagine/proline have been shown to increase the production of embryogenic calli and the regeneration frequency in immature embryo-derived callus cultures of sweet sorghum [156].

To avoid the challenges associated with tissue culture and regeneration, Visarada and colleagues demonstrated the use of floral dip method for *Agrobacterium*-mediated *in planta* transformation of SSV 84 and RSSV 9 genotypes of sweet sorghum to develop transgenic lines resistant to spotted stem borer, *Chilo partellus* [138]. In addition to these, electroporation and pollen-mediated transformation have also been used for transformation in sorghum with little success [157].

### Selection of putative transformants

Genes encoding for selection marker proteins and reporter proteins are usually introduced with T-DNA to select the transformed events. Most common reporter genes used in sorghum include *gfp* that encodes for green fluorescent protein, *uidA* that encodes beta-glucuronidase (GUS), *luc* that encodes for luciferase, and the anthocyanin pigmentation systems R and C1 of maize. The use of selection marker genes and the promoters governing their expression is vital for developing successful transgenic plants [158]. Some of the frequently used selection marker genes in sorghum are *cat* (chloramphenicol acetyltransferase), *npt II* (neomycin phosphotransferase II), *hpt* (hygromycin B phosphotransferase), *bar* (bialaphos resistance), and *manA* (phosphomannose isomerase enzyme). Among these, *bar* is most widely used [158]. Although the most commonly used promoters are *CaMV35S* (Cauliflower Mosaic Virus 35S), maize *adh1* (alcohol dehydrogenase1), maize *ubi1* (Ubiquitin 1), and *actin 1*, ubi1 promoter has been reported to drive the highest level of expression [159].

## QTLs and genes governing biofuel-related traits

Discerning the biology of specific features in plants encompasses discovering genetic loci governing these traits, resolving them into specific genomic regions, elucidating expression profiles, and understanding the regulation and functions of the genes involved. Many agronomic traits of sorghum have been evaluated in this respect.

Correlating genetic units like QTLs to the whole genome can provide information about putative candidates governing specific traits. Mace and colleagues [160] integrated the whole genome sequence information with sorghum QTLs by projecting 771 QTLs onto sorghum consensus map, thereby providing a useful resource for designing efficient strategies for marker-assisted breeding. Later, an atlas of QTLs for biofuel-related traits in sorghum with respect to their chromosomal locations was compiled. It includes 858 biofuel-related QTLs that can be directly used in sweet sorghum breeding to achieve higher yields, more biomass, higher stem soluble sugars on the marginal lands, etc. [161]. A comparative genomic database named The Comparative Saccharinae Genome Resource (CSGR)-QTL has been designed for cross utilization of the information among members of Saccharinae clade and other clades of grasses [162]. The database contains QTL information for *Sorghum, Saccharum, Miscanthus*, and rice. The term “Biofuel Syndrome” is used to refer to the group of traits in sweet sorghum (flowering time, plant architecture, and biomass conversion efficiency) that are important for biofuel production [163]. Below, we summarize the studies that have been carried out to understand the genetic basis of these traits in sweet sorghum.

### Sugar content

Proportion and composition of sugar content in sweet sorghum stalks is a critical factor when considering it as a potential biofuel feedstock. Increased sugar content is reported to be dominant or additive trait. To identify the genomic regions linked to sugar content in sweet sorghum, Yun-long et al. [164] crossed a high sugar content inbred line, early Foger with another inbred line, N32B. Analysis of 207 segregating individuals resulted in identification of two QTLs, which explain total phenotypic variation ranging from 22.2 to 25%. Later, Murray and colleagues [131] evaluated a population derived from sweet sorghum cultivar Rio and grain sorghum cultivar BTx623. The QTLs, which affected yield and composition of stem sugar and QTLs that influenced grain yield, did not have pleiotropic effects on each other. This resulted in identification of several QTLs for sugar components on SBI-01, SBI-02, SBI-03, SBI-05, SBI-06, SBI-07, SBI-10. A novel significant association for brix on chromosome 1 carrying a gene encoding for glucose-6-phosphate isomerase homolog was identified [71]. Shiringani et al. [132] crossed grain sorghum (M71) and sweet sorghum (SS79) and developed a population of 188 recombinant inbred lines (RILs). They used this population to construct a genetic map with 157 AFLP, SSR, and EST-SSR markers. Authors reported 49 significant QTLs associated with sugar-related traits, which include total sugar content, Brix, glucose, and other agronomic traits that affect sugar accumulation like amount/quality of juice, flowering time, biomass (height or stem diameter), and fresh panicle weight. QTL on SBI06 showed significant correlation with majority of the traits, i.e., flowering date, plant height, Brix, sucrose, and sugar content. Lekgari [165] screened RILs from two sorghum lines using SSR markers in four different environments. A total of six Brix QTLs were detected on linkage groups SBI01b, SBI04b, SBI05, and SBI07. These QTLs explain about 6.4–33.9% of phenotypic variation observed in the study. In a recent study, Anami and colleagues [161] reported a total of 38 QTLs for stem brix, twelve for stem glucose, fourteen for stem sucrose, twenty-two for stem sugar, and two for fructose accumulation in sorghum. Rono and colleagues studied the effect of interaction between genotype and its environment on juice and ethanol production [166], whereas Ghate and coworkers analyzed near isogenic lines of sweet sorghum genotype S35 (having stay green loci) and showed that remobilization of sugars occur from stem to grains during drought stress [167]. These studies serve as an important information resource that would be very helpful to dissect the biology of sugar accumulation in sweet sorghum.

Studies on carbohydrate partitioning in sweet sorghum have helped to understand the mechanism involved in source-to-sink movement of soluble sugars. Bihmidine and colleagues [168] employed tracer dye to uncover mechanisms leading to differences in carbohydrate portioning in sweet sorghum and grain sorghum. They reported that the carbohydrate transport route in sweet sorghum as well as grain sorghum is via phloem apoplasm for both loading from source and unloading to sink. Qazi et al. [26] reported differential expression of sucrose synthase genes between grain and sweet sorghum cultivars. They studied the expression of sugar-metabolizing enzymes in sweet sorghum variety SSV 74 in comparison to grain sorghum variety SPV 1616. Expression of sucrose synthase gene *SUC1*, two sucrose phosphate synthases; (*SPS2* and *SPS3*), two sucrose transporter genes; (*SUT1* and *SUT4*); and a vacuolar invertase gene *INV3* was lower in sweet sorghum. Differential expression of sugar-metabolizing enzymes and sugar transporters might have an important role in carbon portioning in sweet sorghum vis-à-vis grain sorghum. However, Bihmidine et al. [168] showed that the sucrose transporter genes *SbSUT2* and *SbSUT4* do not exhibit differential expression in grain and sweet sorghum; thereby, suggesting that all the genes tested by Qazi and coworkers may not be required for the carbohydrate partitioning.

Li and colleagues [169] studied expression patterns of *SbSUT1, SbSUT2, SbSUT3, SbSUT4,* and *SbSUT5* in the stems of sweet sorghum and the heterologous system yeast. Expression in yeast proved that these genes are expressed and translated to functional sucrose transporters. A comparison of sucrose transporter SUT homologs from BTx623 and Rio revealed a difference of nine amino acids. It is most highly expressed in storage tissues like stem and may contribute to enhanced phloem loading and sugar transport to stem in sweet sorghum varieties [129]. Using RNAseq, Mizuno et al. [127] elucidated a comparative expression profile of newly identified sucrose transporter gene family, *SWEET* between sweet sorghum (SIL-05) and grain sorghum (BTx623). Twenty-three *SWEET* genes were identified and implicated in efflux of sucrose from the leaf, unloading sucrose from the phloem in the stem, seed, and pollen development. Recently, another class of sugar transporters “tonoplast sugar transporters” has been suggested to play a significant role in accumulation of sugars in sweet sorghum stems [170].

### Biomass-related traits

Lignin content in cell wall of a plant determines its digestibility and therefore, varieties with reduced lignin content are preferred to produce cellulosic ethanol. Both classical breeding methods and transgenic approaches are being explored to develop varieties with reduced lignin content.

Spontaneous mutations in the genes involved in lignin biosynthesis are associated with a brown color of leaf midrib due to reduced lignin content and are called brown midrib (*bmr*) mutants [171]. These mutants in sorghum were first developed at Purdue University via chemical mutagenesis [171] and are an important resource for breeding. Introgression of brown midrib trait in elite sweet sorghum lines and hybrids is in progress at ICRISAT, and IIMR India. Mutants *bmr*-*1,* -*3,* -*7*, and -*12* are being used for this purpose. The *bmr* parental lines (B/R) will be used to develop elite hybrids (high grain and biomass), which are amenable for lignocellulosic ethanol extraction at lower costs. IIMR has developed SPV 2018, a low lignin and highly digestible brown midrib variety, that has been tested extensively under all India trials and was registered with the National Bureau of Plant Genetic Resources (NBPGR), India in 2015. The introgression of *bmr* genes into elite sweet sorghum lines would result in the development of dual-purpose bioenergy sorghums, which would yield juice for the ethanol production and bagasse for the second-generation biofuel development. The anticipated yields of fermentable sugars from *bmr* sorghum stover upon pretreatment and enzymatic saccharification are expected to be quite high compared to the sorghum stover [172].

Conversely, transgenic sweet sorghum lines having altered lignin content by manipulating the expression of caffeoyl CoA-*O*-methyltransferase (CCoAOMT) have been characterized and patented [154]. Fourteen cinnamyl alcohol dehydrogenase (*CAD*) genes in sorghum genome have been identified; out of which, *SbCAD2* has been shown to play a major role in lignification and is also the target gene in *brown midrib 6* mutants [173, 174].

Plant height is also directly proportional to biomass and is one of the targets in breeding programs with an aim to develop taller cultivars as biofuel feedstock. Murray and colleagues [71] reported three significant associations for plant height on sorghum chromosomes 9 and 6. Another QTL has been identified on linkage group 7 and this region is associated with *Dw3*, a dwarfness allele [131, 165, 175, 176]. Other genetic loci that have been shown to be associated with plant height in *Sorghum bicolor* include *Dw2* on SBI06 [177], *Dw3* on SBI07, and *Dw1* on SBI09 [178]. Including these three, a total of 62 genetic loci conditioning plant height in sorghum have been identified [161]. Further, Yamaguchi and colleagues have shown that *Dw1* reduces cell proliferation activity specifically in stem internodes [179], whereas *Dw1* together with *Dw3* helps in improving lodging resistance [179]. Madhusudhana and colleagues also identified another locus associated with plant height, designated as *Dw4* [180]. *Dw4* shows strong association with blooming habit, a morphological marker. The study also reported five pairs of epistatic QTLs for plant height namely QphA-1/QphI-1, QphA-2/QphD-1, and QphA-3/QphJ-1, which had positive additive/additive interactions and QphA-3/QphI-2, QphE-1/QphI-1, which showed negative epistatic additive/additive effects. Another set of genes that have been found to influence plant height in sorghum include *SbCPS1*, *SbKS1*, *SbKO1,* and *SbKAO1*. These are involved in early steps of GA biosynthesis, and loss of function mutations in any of these genes led to severe dwarfing phenotype [181].

Leaf morphology and root architecture govern radiation usage efficiency, photosynthetic rate, and water/nutrient uptake, which translates into quality of Brix. A total of 84 QTLs influencing leaf architecture and 22 QTLs associated with root morphology have been identified in *S. bicolor* [161]. Fernandez et al. characterized 82 sorghum accessions at genetic level and evaluated these accessions phenotypically for leaf photosynthetic capacity [182]. They analyzed several traits that include carbon assimilation, photochemical quenching, efficiency of energy capture by open PSII reaction centers, transpiration rate, stomatal conductance, and effective quantum yields. This can serve as an important resource to improve carbon assimilation efficiency through breeding programs. Other QTLs of agronomic importance are those associated with main culm height [183], culm length, width and number [184], number of nodes [185], and stem diameter [132, 185, 186].

### Flowering time

Flowering time demarcates the end of vegetative phase and therefore, delayed flowering is desirable for higher biomass accumulation. Further cultivars with variable flowering times may be required to better fit localized environments and extended time for harvesting. Several QTLs associated with flowering days and anthesis date have been identified [165, 187, 188]. In addition, several loci (*Ma1*-*Ma6*) that control the photoperiod sensitivity/maturity in sorghum have been identified [184, 189, 190]. *Ma1* codes for a flowering repressor *SbPRR37,* which is circadian clock-regulated and represses flowering during long days. It played a critical role in early domestication of sorghum. Similarly, *Ma6,* encoded by *SbGhd7*, has been characterized as repressor of *EARLY HEADING DATE 1 (SbEHD1*) and reported to suppress flowering in sorghum during long days [190]. *Ma3* (Sb01g037340) encodes *PHYTOCHROME B* (*PhyB*), a red-light photoreceptor that plays an important role in photoperiod sensing. Upon sensing the light signal, it represses the expression of *TB1 (Teosinte Branched 1*) gene and *DRM1* (dormancy-associated gene) thereby, resulting in axillary bud outgrowth [177, 189]. Similarly, *Ma2*, *Ma4*, and *Ma5* are also associated with photoperiod sensitivity in sorghum. Calvino and colleagues [191] have identified cluster of miRNA169 on chromosome 1 and another on chromosome 7. Chromosomal segments having miRNA169 clusters show significant synteny with the chromosomal segments carrying linked bHLH and CONSTANS-LIKE genes from both monocot and dicot species. It suggests a strong conservation among flowering and plant height-related genes and miRNAs that accounts to a certain extent, for the linkage drag observed in drought and flowering traits [191].

## Biotic and abiotic stress response

Biotic and abiotic stresses adversely impact the crop productivity and traits important for biofuel production. Therefore, adaptation and tolerance towards abiotic and biotic stresses is critical for the survival of a plant under suboptimal conditions. Anami and colleagues [163] have recently reviewed the key biotic and abiotic stresses that impact sorghum crop. Authors have listed a comprehensive list of 350 QTLs related to biotic and abiotic stress tolerance in sorghum. Further, they have highlighted drought stress as major cause for limiting sorghum potential in tropical regions, whereas in temperate environments, early season cold stress is the major constraint.

Hufnagel and coworkers investigated the role of homologs of *OsPSTOL1* in the response of *S. bicolor* to low phosphorous [192]. Results clearly suggested an important role of *SbPSTOL1* in reducing root diameter leading to enhanced phosphorous uptake under low concentration in hydroponics. On the other hand, *Sb03g006765* and *Sb03g0031680* alleles were linked to increasing root surface area and increased grain yield in a low-phosphorous soil. *SbPSTOL1* genes co-localized with QTL for traits underlying root morphology and dry weight accumulation under low P via linkage mapping. We have recently performed a comprehensive analysis of TCP proteins in sorghum and prioritized sorghum TCP proteins important for governing the plant architecture and abiotic stress tolerance [192].

Due to high levels of sugars accumulated in the stalks, sweet sorghum attracts several insect pests that can take heavy toll on overall production. Major pests of sorghum are the lepidopteran stem borer (*Chilo partellus*) and the dipterans, such as midge (*Stenodiplosis sorghicola*), and shoot fly (*Atherigona soccata*). The pests, which specifically affect sweet sorghum and its sugar accumulation, are sorghum midge and midrib panicle-feeding bugs (head bugs) like *Eurystylus oldi* Poppius. Recently, Harris-Shultz and coworkers identified a major QTL associated with number of eggs of southern root-knot nematode (*Meloidogyne incognita*) in sweet sorghum [193]. Such regions can be used to engineer insect resistance in sorghum. Sorghum plants produce two antimicrobial compounds (luteolinidin and apigeninidin), known as phytoalexins that help plants to protect themselves from pathogens [194]. Further characterization of their biosynthetic pathways and mechanism of action will help to utilize these chemicals to induce pathogen resistance in Sorghum.

## Conclusions

Sweet sorghum, with its array of adaptive features and low input requirements, is one of the leading candidates for biofuel feedstock. It has potential to solve two major issues. Firstly, it can play a significant role in addressing the growing need for renewable energy to displace fossil fuel-based energy resources. Secondly, instead of competing with food crops for arable land, it will rather help in conservation of marginal lands by converting them to agricultural land. However, Sorghum exhibits huge genetic diversity and resources towards region-specific climatic conditions or changing climatic conditions, and amount of fermentable sugars and grain yields vary considerably in different sweet sorghum cultivars. Therefore, screening and selection of appropriate varieties for each region is critical for optimum results.

Also, response of sweet sorghum cultivars towards region-specific climatic conditions or changing climatic conditions is a critical aspect for large-scale cultivation. Usually, grain yield in sweet sorghum is very low and grains are not suitable for use as human food.

Unexpected yield losses due to environmental stresses and disease outbreaks is another major concern on large-scale planting at marginal lands. A significant number of studies have been initiated to understand the mechanism of disease resistance and abiotic stress tolerance in sorghum. However, most of the studies reported till date have focused on single stress, whereas under natural environment conditions, a plant is simultaneously subjected to multiple stress factors [195] and the corresponding response is different compared to a single stress. Studies elucidating the mechanism behind combined stress responses mimicking real-life situation in the fields would be needed to optimize breeding programs and agronomic practices required under different climatic conditions.

Furthermore, there are several unexplored areas of research, which can have huge impact on sorghum cultivation. Efforts to develop multipurpose sweet sorghum cultivars with high sugar as well as grain yields have been initiated using both classical and biotechnological approaches to make it economically more attractive. In-depth sequencing of whole genome of a sweet sorghum cultivar is highly awaited to assist in gene discovery and to initiate genome-wide association studies.

## Abbreviations

AICRP: All India Coordinated Research Project
AFLP: amplified fragment length polymorphism
*adh1*: alcohol dehydrogenase1
*bar*: bialaphos resistance
*bmr*: brown midrib
*CAD*: cinnamyl alcohol dehydrogenase
*cat*: chloramphenicol acetyltransferase
*CaMV35S*: cauliflower mosaic virus 35S
CCoAOMT: caffeoyl CoA-*O*-methyltransferase
CSGR: Comparative Saccharinae Genome Resource
DArTs: Diversity Array Technology
*DRM1*: dormancy-associated gene 1
EST: expressed sequence tag
GRIN: Germplasm Resources Information Network
*hpt*: hygromycin B phosphotransferase
ICAR: Indian Council of Agricultural Research
ICRISAT: International Crops Research Institute for Semi-Arid Tropics
IIMR: Indian Institute of Millets Research
JGI: Joint Genome Institute
LS: Linsmaier & Skoog
*manA*: phosphomannose isomerase enzyme
MS: Murashige and Skoog
NARI: Nimbkar Agricultural Research Institute
NBPGR: National Bureau of Plant Genetic Resources
*npt II*: neomycin phosphotransferase II
QTL: quantitative trait loci
RFLP: restriction fragment length polymorphism
SNP: single-nucleotide polymorphism
Sorghum FDB: Sorghum Functional Genomics Database
SorGSD: Sorghum Genome SNP Database
*SPS*: sucrose phosphate synthases
STS: sequence-tagged sites
SSR: simple sequence repeats
*SUT*: sucrose transporters
*TB1*: Teosinte Branched 1
*ubi1*: Ubiquitin 1
USDA: United States Department of Agriculture
